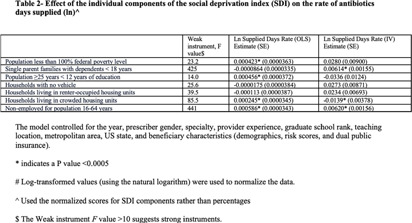# The Impact of Deprivation on Outpatient Medicare Part D Antibiotic Prescription in the US Using Instrumental Variable Approach

**DOI:** 10.1017/ash.2025.339

**Published:** 2025-09-24

**Authors:** Mayar Al Mohaje, David Slusky, David Nix, Catia Nicodemo

**Affiliations:** 1 Baylor College of Medicine; 2 University of Kansas; 3 University of Arizona College of Pharmacy

## Abstract

**Background:** Several studies have shown an association between deprivation and excessive antibiotic use in the US. However, these studies were limited by their geographic design, making them unable to assess a causative relationship. This study analyzed the impact of socioeconomic deprivation on antibiotic days supplied among older Medicare Part D beneficiaries in the US using an instrument variable (IV) approach. **Method:** This study utilized the Medicare Part D and the Social Deprivation Index (SDI) repositories. The maximum Earned Income Tax Credit was chosen as an IV to consider the reverse causality of the SDI values. Spatial dependence between predicted SDI and study outcome (log antibiotic days supplied per 100 beneficiaries) was evaluated by global Moran’s I analysis and cluster mapping. Linear regression models were performed to assess the impact of predicted SDI or its components (poverty, single parent, low education, no car, renter-occupied, crowding, and non-employment) on the study outcome. The study adjusted for the following confounders: prescriber gender, specialty, graduate school rank, teaching location, metropolitan area, US state, and beneficiary characteristics (demographics, risk scores, and dual public insurance). **Results:** A total of 438,431 providers were included. There was no spatial dependence between the predicted SDI and study outcome (I = 0.007, P = 0.0656, Figure). Higher predicted SDI values resulted in higher antibiotic days supplied (log) per 100 beneficiaries (estimate 0.58, SE 0.16, P) **Conclusion:** This study showed a causative relationship between SDI and antibiotic days supplied. It highlights opportunities for public health in the US to explore gaps in antimicrobial stewardship. More studies are needed to investigate the knowledge and attitudes of patients and providers and potential barriers to access that might impact antibiotic prescription in older patients.

**Figure:**
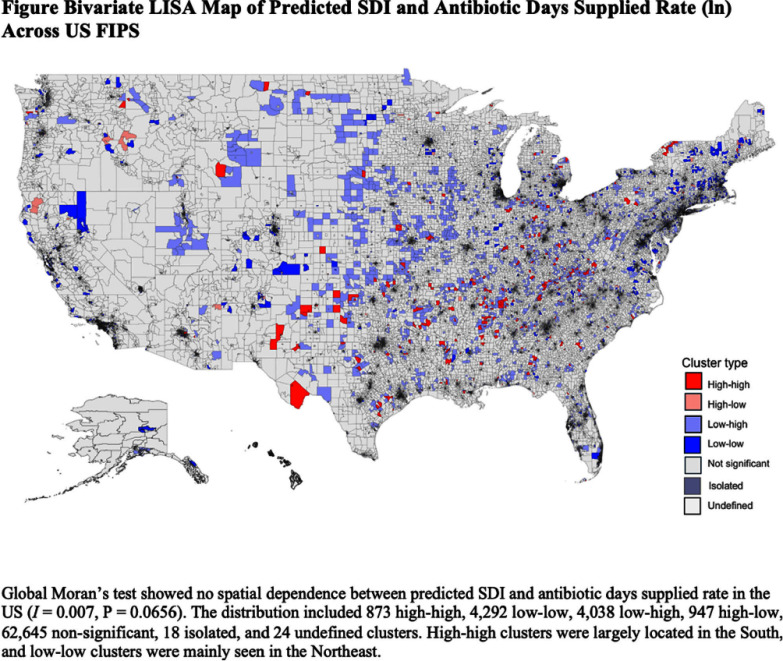

**Figure:**
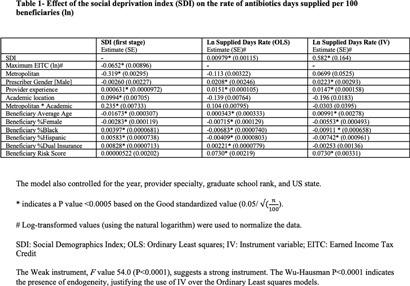

**Figure:**